# 

**Published:** 1894-12-29

**Authors:** 


### CONTENTS.

**Burning Questions** - 215

**Channels of Infection** - 216

**Therapeutics and the Lesser Circulation.** By Sir B. W. RICHARDSON, M.D., F.R.S. II. - 217

**Studies in Applied Sociology.**—"Where the Silky Cotton Grows" - 218

**Medical Progress and Hospital Clinics:**

Malignant Growths - 221

On Hemiplegia - 222

PROGRESS IN SURGERY—General Surgery - 222

PROGRESS IN MEDICINE—Infectious Diseases - 223

NEW DRUGS AND PREPARATIONS - 226

**Annotations.**—Sane Persons in Private Asylums—The Clinical Society: Discussion on Antitoxin—The Electrolytic Treatment of Pruritus - 219

**Notes and News** - 220

**The Institutional Workshop:**

AN INSTITUTION FOR INEBRIATES.—Walnut Lodge Hospital Hartford, Ct. - 227

PRACTICAL DEPARTMENTS.—Bedsteads and Bedding - 227

HOSPITAL FESTIVALS AND MEETINGS - 228

**Editor's Letter Box.**—The Power of the General Medical Council 230

**Scraps and Gleanings** - 230

**The Book World of Medicine and Science** - 231

### EXTRA SUPPLEMENT.—THE NURSING MIRROR.

**News from the Nursing World.**—Our Princess—A Happy New Year—Guy's Hospital Nurses—"A Necessity and a Luxury"—Santa Claus Society—Who Makes the Beds?—False or True?—Unfed and Unnursed—Where was the Thermometer?—St. Elizabeth's Home—Private Patients in Africa—A Noble New Year's Gift—Short Items - xci

**The Dietetic Management of Infancy.** III.—Feeding Bottles and Food - xciii

**Our Christmas Competitions** - xciv

**Entertainments** - xciv

**Our American Letter** - xcv

**Notes from St. Helena** - xcv

**Private Nursing at Home and Abroad** - xcvi

**"Truth" Toy Competition** - xcvi

**Everybody's Opinion** - xcvii

**Nursing in Holland** - xcvii

**Christmas Books—For Reading to the Sick** - xcviii

**Dress and Uniforms—Presentations** - c

